# Tardive Dyskinesia After Aripiprazole Treatment That Improved With Tetrabenazine, Clozapine, and Botulinum Toxin

**DOI:** 10.3389/fphar.2019.00281

**Published:** 2019-03-20

**Authors:** Lourdes Aguilar, Carolina Lorenzo, Raquel Fernández-Ovejero, Carlos Roncero, Angel L. Montejo

**Affiliations:** ^1^Psychiatry Service, Health Care Complex, Salamanca, Spain; ^2^Institute of Biomedical Research of Salamanca (IBSAL), Salamanca, Spain; ^3^Department of Psychiatry, University of Salamanca, Salamanca, Spain; ^4^Department of Psychiatry, Navarra Hospital Complex, Pamplona, Spain; ^5^Nursing School E.U.E.F., University of Salamanca, Salamanca, Spain

**Keywords:** aripiprazole, tardive dyskinesia, botulinum toxin, antipsychotics, adverse effects

## Abstract

We report on a patient with tardive dyskinesia (TDK) treated with aripiprazole, a third-generation antipsychotic with partial D2 agonist-antagonist activity at both the dopamine and serotonin receptors. The patient’s condition improved with administration of a combination of tetrabenazine, botulinum toxin, and clozapine, which has previously not been used. We suggest that this treatment combination may have potential benefits for patients with TDK. After aripiprazole discontinuation, the patient was treated with clozapine (150 mg/day) and biperiden (8 mg/day). Due to a lack of improvement, we administered 300 units (intramuscularly; IM) of botulinum toxin into the paravertebral muscles every 3 months and 1,000 units IM every 4 months in addition to tetrabenazine (75 mg/day) and biperiden (8 mg/day). The patient stopped this treatment, at which point TDK reappeared. After starting a treatment regimen of clozapine (100 mg/day), tetrabenazine (75 mg/day), and botulinum toxin (300 units IM), the patient’s symptoms remitted.

## Introduction

Little is known about the treatment of tardive dyskinesia (TDK) caused by aripiprazole. This antipsychotic is associated with a lower theoretical risk of TDK compared with classical neuroleptics. Several publications have even presented clinical cases that showed an improvement in dyskinesia or dystonia after switching to aripiprazole. However, data published since the commercialization of aripiprazole suggest that the risk of TDK could be higher than initially thought.

The relatively low risk of TDK due to aripiprazole is attributed to the unique partial agonistic activity of aripiprazole at the D2 receptor (Margolese et al., 2005). However, aripiprazole has one of the highest D2 receptor affinities at a therapeutic dose of all antipsychotic drugs. Theoretically, long-term exposure of these receptors to aripiprazole could cause irreversible hypersensitivity.

## Methods

We describe the case of a 23 year-old woman who developed tardive dyskinesia after taking aripiprazole for 6 years which was successfully treated with a combination of tetrabenazine, botulinum toxin, and clozapine. A thorough literature search was performed using PubMed, Medline, Ovid, Embase, and Google Scholar about the existence of other possible cases treated with the same drug combination without finding similar cases including the International Medical Case Reports Journal.

A written informed consent was obtained from the patient for the publication of this case report.

## Case Presentation

### Presenting Features

She presented with progressive lateral left deviation of the trunk while walking, which was secondary to dystonic contraction of her paravertebral muscles. The patient was first treated during her first psychotic episode in October 2000. She had just finished high school and was preparing for her university entrance exams. She lived with her parents and older sister. Her family explained that her behavior was strange and introverted. She did not speak, was depressed, and experienced hallucinations.

### Progression, Treatment, Follow-Up, and Outcome

The patient received olanzapine between October 2000 and February 2001 and was followed monthly at our outpatient clinic in Salamanca, Spain. Her symptoms improved substantially. Afterward, she continued treatment with olanzapine at a dose of 10 mg/day and fluoxetine at a dose of 20 mg/day. Her family felt that she was improving. The patient started her university education in economics and participated in social events with friends. The patient’s laboratory samples were normal. Her prolactin level increased mildly, to 39 ng/ml (normal range: <25 ng/ml), which was of no clinical relevance, and her psychotic symptoms progressively improved.

After 1 year, the patient’s olanzapine dose was decreased to 5 mg/day. The patient’s psychotic symptoms reappeared in February 2004, and she was enrolled into an open prospective clinical trial with aripiprazole alone at a dose of 15 mg/day. The patient’s psychotic symptoms improved markedly while on aripiprazole. After 2 months, her creatine phosphokinase level increased to 554 μg/l (normal range: 10–120 μg/l), which was considered an adverse event of no clinical relevance.

The patient continued to have delusional thoughts. In September 2004, her dose of aripiprazole was increased to 25 mg/day. The patient’s laboratory samples were normal. Her prolactin level was normal, at 16 ng/ml (normal range: 2.8–25 ng/ml). In 2007 and 2008, the patient’s delusions disappeared, but she spent most of her time alone in her room. The patient had serious concerns about her professional future. In 2009, she stopped attending the outpatient unit.

In August 2010, the patient started landing toward one side and the back when walking. She developed severe dyskinesia with a score of 12 on the Abnormal Involuntary Movement Scale (AIMS). At this point, she reported hearing good voices that helped her and thought that they came from God. She started to write verses of poetry that made no sense.

Due to her abnormal walking movements, the aripiprazole dose was slowly decreased from 25 mg/day to 10 mg/day. In June 2011, the patient’s magnetic resonance and computed tomography scans were normal. Aripiprazole treatment was discontinued, and the patient started tetrabenazine treatment at a dose of 100 mg/day.

In October 2011, the patient’s parents divorced, and she lived with her mother. As she continued to hear voices that she believed were coming from God, treatment with clozapine (150 mg/day) and biperiden (8 mg/day) was started. This regimen provided a partial response. Due to a lack of improvement in her TDK, she was treated with 300 units IM of botulinum toxin every 3 months (December 2011 to July 2012) and 1,000 units IM every 4 months (October 2012 to October 2013) in addition to the tetrabenazine (75 mg/day) and biperiden (8 mg/day).

In December 2012, the patient stopped all treatment and her symptoms worsened. We then began another course of treatment that included botulinum toxin (300 units IM) and tetrabenazine (100 mg/day), but she did not improve. Thus, we added clozapine (100 mg/day), at which point she improved significantly. Some dyskinetic symptoms remained, but she walked more safely and faster without landing movements. The patient’s psychotic symptoms also improved, and she reported feeling much more motivated and less isolated.

The patient stopped this treatment, and her TDK and psychotic symptoms reappeared. We restarted treatment with clozapine (100 mg/day), tetrabenazine (75 mg/day), and botulinum toxin (300 units IM), and her TDK and psychotic symptoms improved.

### Discussion and Management Strategies

#### Tetrabenazine

Tetrabenazine is used in the treatment of hyperkinetic movement disorders, particularly TDK and chorea. It has been known since the seventies, but it was only recently introduced into the market (Tarsy et al., 2011). Tetrabenazine can be used both in the pediatric population and in adults with good tolerance (Chen et al., 2012).

The main essential pharmacological property of tetrabenazine is the reduction of monoamines (dopamine, serotonin, and noradrenaline) in the CNS, particularly dopamine, and it causes a reversible inhibition of the activity of vesicle monoamine transporter 2 (Guay, 2010). With regard to Tourette’s syndrome, nine out of eleven studies (controlled prospective and retrospective studies) showed good results for phonic and motor tics in pediatric and adult patients (Guay, 2010).

The adverse effects of tetrabenazine are: depression, fatigue, parkinsonism and somnolence. In our case, the patient presented depression and fatigue.

#### Botulinum Toxin

Botulinum toxin has been successfully used in ophthalmological and neurological processes that involved an increase of the muscle tone. It has a paralyzing action that binds to the presynaptic nerve terminals; it is then internalized and inhibits the secretion of acetylcholine. The subtypes of botulinum toxin that are related to pathologies in humans are type A, B, and E. The most widely used subtype in clinical practice is subtype A (Chatterjee et al., 1997).

Botulinum toxin is used in cases of TDK with good results. Patients with involuntary tongue protrusion have showed an improvement with a treatment involving injections into the genioglossal area, although some authors have described secondary effects to the treatment, including episodes of dysphagia and dysarthria with high doses (van Harten and Hovestadt, 2006).

The toxin was also used for the management of idiopathic cervical dystonia in 205 patients by Jankovic and Schawartz (1990) who proved its efficacy. Its use was analyzed over 5 years and they did not find any adverse effect or the appearance of resistance to the treatment.

With regard to the use of *biperiden* for the treatment of TDK, Lamberti et al. (2017) published the case of a 13-year-old patient who was referred to their unit for depression and behavior disorders. She started treatment with aripiprazole at 7.5 mg/day, but after 3 weeks she displayed involuntary movements of her mouth and slurred speech. The treatment with aripiprazole was suspended and she was treated with clonazepam 4 mg/day and an antioxidant (vitamin E), without results. Then, treatment was started with biperiden 2 mg/day, and 3 weeks later the TDK had improved (Lamberti et al., 2017).

#### Clozapine

Clozapine was first used in the treatment of aripiprazole-induced TDK on a 24-year-old man with bipolar disorder who had been given 15 mg aripiprazole. 3 years later he presented dystonia and hyperextension of the neck, particularly on the right side. The symptoms disappeared with the administration of clozapine 100 mg/day (Joe et al., 2015). Some of the risk factors that have been described for TDK in patients with affective disorders include interaction between antipsychotics and antidepressants and the use of high doses of antipsychotics over a short period of time (Klawans and Rubovits, 1972; Casey, 1988; Louza and Bassitt, 2005; Skidmore and Reich, 2005; Matsuda et al., 2012). This patient benefited twice from the treatment with clozapine. On the one hand, it treated the manic symptoms and, on the other hand, his TDK. Also, the patient was young, and the duration of the TDK was short (8 months), which contributed to a good response to the treatment (Hazari et al., 2013).

Unlike that case, in our patient there was a relatively long period between the suppression of aripiprazole and the administration of clozapine, which supports the hypothesis that clozapine has therapeutic effects on patients with aripiprazole-induced TDK. Even though clozapine has some limitations (it can cause agranulocytosis), it can be an effective alternative in the treatment of irreversible TDK.

Previous studies (Lungu et al., 2009; Friedman, 2010) have described cases of aripiprazole-induced TDK. In one of those cases, the symptoms remitted after removal of aripiprazole (Skidmore and Reich, 2005), but in other cases it was necessary to use trihexyphenidyl, botulinum toxin and deep brain stimulation for the symptoms to improve. That is, in some cases, removal of aripiprazole, by itself, is not enough to reverse TDK.

The physiopathology of TDK is still unknown. It is believed to be due to hypersensitivity of the D2 receptors after being blocked over a long period of time (Hazari et al., 2013). According to Aquino and Lang (2014), there is a balance between the direct and indirect pathways of the basal ganglia. The activation of the direct pathway leads to the appearance of movements, whereas the activation of the indirect pathway causes a decrease in the velocity and amplitude of the movements. The hypersensitivity of the D2 receptors may be the cause of hyperkinesia (Aquino and Lang, 2014). Another theory on the origin of TDK is that D2 receptors being blocked over a long period of time create a lack of adaptation of the synaptic plasticity in the corticostriatal transmission and oxidative stress (Teo et al., 2012; Cho and Lee, 2013).

It is believed that the advantage of clozapine and quetiapine of having a low risk of producing TDK is due to their role as blockers of 5HT2A and 5HT2C, which increases the release of dopamine (Meltzer, 2013). It is also thought that a prolonged inhibition of the D2 receptors can result in the sensitization of the D1 receptors and create an imbalance in the striatum. If we take into account this last hypothesis, D1 antagonists may be a possible treatment for TDK (Trugman et al., 1994). Clozapine has a high affinity for D1 receptors and a relatively low affinity for D2 receptors (Nordström et al., 1995). Another difference between clozapine and aripiprazole is the fact that this last substance is a partial antagonist of 5HT2A and 5HT2C receptors. This could explain why clozapine can reverse aripiprazole-induced TDK (Bolden et al., 1991). The probabilities of aripiprazole causing TDK are low due to its role as a partial D2 receptor agonist (Margolese et al., 2005).

Patra (2016) describes two cases of TDK associated with the consumption of aripiprazole. One of the cases was observed in a young adult treated with aripiprazole and fluoxetine. In the second case, TDK appeared after the removal of aripiprazole in a middle-aged woman with affective disorder. Both patients showed movements of the mouth, jaw and tongue. In the first case, the appearance of TDK may be explained by the inhibitory action of fluoxetine on CYTP4502D6, which may have increased the plasma levels of aripiprazole. Fluoxetine may also have raised the serotonin levels, which would in turn raise the dopamine levels in the brain, triggering TDK (Leo, 1996). According to the recommendations of evidence-based medicine, a decrease in the dose of aripiprazole is necessary in the treatment of TDK (Bhidayasiri et al., 2013).

In the second case, the removal of aripiprazole is considered to be the cause of the dyskinesia. Being a woman, having a history of treatment with other neuroleptics and having an affective disorder are considered risk factors for TDK (Schwartz and Raza, 2008; Peña et al., 2011).

Another case, published by Edwige et al. (2016), describes a 74-year-old man who was treated for an episode of depression with escitalopram (10 mg/day) and aripiprazole (5 mg/day), although no delirium or melancholic symptoms were observed, which means that the combination seemed inappropriate. After 9 months of treatment, aripiprazole was suspended for dyskinesia of mouth, tongue and face and chorea in the limbs. The abnormal movements persisted in spite of the removal of aripiprazole. The patient was treated with tetrabenazine (37.5 mg/day), clonazepam (0.6 mg/day), and baclofen (10 mg/day). The patient refused to start treatment with clozapine.

The blocking role of 80% of all D2 receptors by aripiprazole improves the psychotic symptoms but increases the risk of motor side effects (Woods et al., 2010). Mamo et al. (2007) claim that 80% occupancy of the striatal D2 receptors can be achieved with 10 mg of aripiprazole. Extrapyramidal movements have been observed with occupancy of over 90% of all D2 receptors (Mamo et al., 2007). Murphy et al. (2016) published that 5 mg of aripiprazole blocked 55% of all the D2 receptors in the striatum, whereas 6 mg blocked 77% of those receptors and 51% of all the D2 receptors in the frontal lobe. Low doses of aripiprazole (2–5 mg) have been associated with a higher blocking of extra-striatal D2 receptors than striatal D2 receptors (Kegeles et al., 2008). Peña et al. (2011) published a case report of a patient with low doses of aripiprazole (5 mg/day) who presented TDK.

## Conclusion

The aim of this report was to draw attention to the treatment of TDK in a patient who was treated with aripiprazole, a third-generation antipsychotic with partial D2 agonist- antagonist action at both the dopamine and serotonin receptors. This patient improved with a combination of tetrabenazine, botulinum toxin, and clozapine. This combination of drugs has previously not been used. We suggest that this drug combination may be beneficial for patients with TDK.

## Author Contributions

LM and AM performed the review, wrote the manuscript, and incorporated feedback from all co-authors. CL, RF-O, and CR had substantially contributed to drafting the article or revising it critically for important intellectual content. All authors had given the final approval of the version to be published.

## Conflict of Interest Statement

AM has received consultancy fees or honoraria/research grants in the last 5 years from Eli Lilly, Forum Pharmaceuticals, Rovi, Servier, Lundbeck, Otsuka, Janssen Cilag, Pfizer, Roche, Instituto de Salud Carlos III, Junta de Castilla y León, and Osakidetza. CR has received fees to give lectures for Janssen-Cilag, Ferrer-Brainfarma, Pfizer, Indivior, Lundbeck, Otsuka, Servier, GSK, Rovi, Astra, Gilead, MSD, Sanofi, and Exeltis. He has received financial compensation for his participation as a board member of the Janssen-Cilag, Lundbeck, Gilead, MSD, Mundipharm, INDIVIOR, Exceltisand Martindale board. He has carried out the PROTEUS project, which was funded by a grant from Reckitt Benckiser/Indivior. He received two medical education grants by Gilead. CL received consultancy fees or honoraria/research grants in the last 5 years from Janssen-Cilag. The remaining authors declare that the research was conducted in the absence of any commercial or financial relationships that could be construed as a potential conflict of interest.
